# Improving competencies and skills across clinical contexts of care: a qualitative study on Malawian nurses' experiences in an institutional health and training programme

**DOI:** 10.1002/nop2.1030

**Published:** 2021-08-06

**Authors:** Camilla Grøver Aukrust, Patrick Dongosolo Kamalo, Ruth Jane Prince, Johanne Sundby, Chimwemwe Mula, Lucinda Manda‐Taylor

**Affiliations:** ^1^ Department of Community Medicine and Global Health Institute of Health and Society Faculty of Medicine University of Oslo Oslo Norway; ^2^ Department of Neurosurgery Oslo University Hospital Oslo Norway; ^3^ Department of Surgery Elizabeth Central Hospital Ministry of Health Blantyre Malawi; ^4^ Kamuzu College of Nursing Clinical Nursing Department University of Malawi Blantyre Malawi; ^5^ Department of Health Systems and Policy School of Public Health and Family Medicine College of Medicine University of Malawi Blantyre Malawi

**Keywords:** competency, education, health education, malawi, neurosurgery, nurse practitioners

## Abstract

**Aim:**

To explore what competencies and skills Malawian nurses gained after participating in an institutional health and training programme in Norway and how they viewed these competencies applicable upon return to Malawi. Furthermore, to examine facilitators and challenges experienced on the exchange programme and opportunities and obstacles to make the competencies usable in own local hospital context.

**Design:**

Qualitative study with an explorative design.

**Methods:**

Fourteen interviews and one focus group discussion were conducted at Queen Elizabeth Central Hospital, Blantyre, Malawi, from August to September 2018.

**Results:**

Competencies gained in Norway included clinical skills, teamwork, coordination and strengthened professionalism. The main finding was that the exchange programme was a transformative experience. Upon return to Malawi, the competencies gained on the exchange were helpful. However, the return was characterized by mixed emotions due to the considerable difference between the two clinical settings.

## 1 INTRODUCTION

With a deficit of over 600,000 nurses in sub‐Saharan Africa, most countries in the region still concentrate on basic nursing training, leaving very little room for specialized or advanced nursing (Bialous & Baltzell, 2020; Kinfu et al., 2009). As a result, some hospitals have resorted to institutional health partnerships as a way of improving competencies in specialist nursing care, like oncology, critical care and paediatric nursing (Bentounsi & Nazir, 2020; Hockenberry et al., 2020; Ruthe & North, 2020). Institutional partnerships aim to build workforce capacity and sustainable health institutions to improve health systems, health services and patient outcomes (Kelly et al., 2015).

## 2 BACKGROUND

Like other surgical disciplines, neurosurgery remains scarce in sub‐Saharan Africa, with some populations having more than 10 million people for one neurosurgeon (Fuller et al., 2016). The sub‐Saharan African country Malawi has a population of about 18 million. In 2011, one of Malawi's physicians returned to Blantyre and began working at the tertiary referral centre, Queen Elizabeth Central Hospital (QECH), after completing neurosurgical specialist training abroad (Gadama, 2018). As the only neurosurgeon in the country at that time, several challenges existed. The lack of medical equipment, a dedicated ward for neurosurgical patients, and limited capacity and competency related to neurosurgical caretaking within the nursing cadre were some of the challenges. Because of this, and on initiation from Malawi, the Blantyre‐Oslo Neurosurgery Exchange Program (BONEP), a long‐term institutional health partnership, primarily concerned with improving healthcare providers' competencies, was established in 2013. The main partners involved in BONEP are Norway/Oslo University Hospital (OUH), the Norwegian Agency for Exchange Cooperation (NOREC, which lies under the Norwegian Ministry of Foreign Affairs), the Norwegian Embassy in Malawi, the Ministry of Health in Malawi through QECH and the College of Medicine in Blantyre.

BONEP's main intention is to establish a sustainable neurosurgical service for Malawi's population by training doctors, ward and theatre nurses, anaesthetic clinical officers, theatre assistants and biomedical technicians (Bandlien & Palm, 2016; Gadama, 2018). Furthermore, to address Malawi's challenges in providing neurosurgical care, especially at QECH, a seven‐bed, high‐dependency neurosurgical unit designated for preoperative and postoperative management of neurosurgical patients was built. In addition, medical supplies and technologies were transferred from Norway to Malawi, including modern neurosurgery operating equipment such as bipolar diathermy, microscopes and high‐speed drills. Lastly, a training programme was developed, involving local on the job training by staff from Norway, experienced in neurosurgical care, who came to stay in Malawi and a 6‐month job exchange of healthcare providers from Malawi to Norway (Bandlien & Palm, 2016; Gadama, 2018).

A prominent feature of the health partnership philosophy is that sharing competencies and knowledge can diminish global inequalities within health and education. This perspective rests on the foundation that despite differences relating to culture, competence and clinical context of care, there is also a fundament for mutual learning and collaboration (Frenk et al., 2010). A theoretical concept that describes how learners enter new contexts and may acquire new competencies within a shared community of practice is Lave and Wenger's theory on situated learning (Lave & Wenger, 1991). In addition, the concept of communities of practice has increasingly been applied in health‐related fields (Cruess et al., 2018).

Many definitions of competency have been proposed in the medical literature since the 1970s, and most are complex (Carraccio et al., 2002). We applied the broad definition of professional medical competence offered by Epstein and Hundert, which defines it as ‘the habitual and judicious use of communication, knowledge, technical skills, clinical reasoning, emotions, values, and reflection in daily practice for the benefit of the individual and the community being served’ (Epstein & Hundert, 2002, p. 226). Epstein and Hundert further state that professional competence is dependent on the relationship between the know‐how of the person; the task that needs to be performed in the world; and the ‘ecology of the health systems and clinical contexts in which those tasks occur’ (Epstein & Hundert, 2002, p. 228). We find this definition purposeful, as it emphasizes the comprehensiveness of professional competence and addresses the significance of context, an essential element of what this study investigated. Pragmatically, health workers' competencies refer to set procedures and processes that providers in a given speciality need to be conversant and skilled in (Nursalam et al., 2020).

### 2.1 Research questions

Nursing care in Malawi is practised in a context of a chronic shortage of staff, medical stuff and supplies, space and systems, often referred to as the four S theory (Farmer, 2014). The Malawian context, therefore, differs dramatically from the Norwegian context. An essential part of BONEP includes the 6‐month exchange of Malawian nurses to Norway/OUH. Therefore, this study's main research question was: what competencies do Malawian nurses report to have gained during the job exchange in Norway? More specifically, we aimed to answer the following questions: what do Malawian nurses describe as facilitators and challenges on exchange in Norway? How do Malawian nurses consider the competencies gained at OUH to be valuable and applicable in a Malawian urban public hospital context? Besides, we aimed to answer the question, what do the Malawian nurses identify as opportunities and obstacles in making the competencies gained in Norway helpful in a local clinical context?

## 3 THE STUDY

### 3.1 Study design and study setting

We conducted a qualitative study with an explorative design. The first author engaged in data collection from August to September 2018 in QECH in Blantyre, Malawi, using in‐depth interviews and focus group discussion. QECH is one of four central hospitals in Malawi and offers specialized treatment at the tertiary level (Henry et al., 2015). With its 1,000 beds, QECH serves as the main hospital for Blantyre and its vicinity (around 800,000 population). Also, it serves as the main referral hospital for the Southern region of Malawi (approximately 7,750,000 population) (Morton et al., 2020).

### 3.2 Method

The trustworthiness and rigour in collecting the data were maintained by ensuring that the nurses who participated in the BONEP exchange programme were willing to candidly share their experiences through semi‐structured, open‐ended, in‐depth interviews and the focus group discussion. The different interview methods helped produce a holistic set of data. In addition, the researcher conducted repeated visits of the audio‐recorded interviews to check emerging themes and remain faithful to the participant's accounts on the enabling and challenging factors of improving Malawian nurses' competencies and skills across clinical contexts of care. The first author discussed the emerging themes with the supervisor with qualitative research expertise (LMT). Any assumptions about the findings and themes were discussed or challenged until we reached a consensus on the final themes. The main findings were supported by the use of verbatim extracts from the participants (Noble & Smith, 2015).

### 3.3 Study population, inclusion and exclusion criteria, sampling and sample size

The study population consisted of nurses working in the intensive care unit, the neurosurgical operating theatre, the neurosurgical high‐dependency unit and the paediatric neurosurgical ward in QECH. Inclusion criteria were that the nurses had been on exchange to Norway through BONEP and worked with patients' pre‐, per‐ or postoperatively in one of the above mentioned clinical wards, both before and after the exchange period in Norway. Having moved on to further education or other positions in the hospital were not considered an exclusion criterion. In addition, healthcare providers of other professions than nursing, or nurses who had not been on exchange to Norway, were excluded.

We used convenient sampling to identify relevant participants (Maxwell, 2012). Applying the concept of maximum variation (Maxwell, 2012), we included participants from different wards, of both genders, unlike years of exchange in Norway (from 2013–2018) and, therefore, different length of stay in Malawi after the 6‐month exchange. Ward nursing managers were asked to inform their staff that the researcher would invite them to participate in the study. All the participants were known to the data collector (first author) since they had all been to Oslo, where the researcher works. They were recruited in the hospital area at QECH.

By the time we were conducting the study, 20 nurses had been on exchange to Norway through BONEP. We had intended to invite all of them to participate. However, four nurses were not available in the hospital, and two nurses accepted but could not find a convenient time for interviews. Thus, a total of 14 in‐depth interviews were conducted. After the in‐depth interviews had been carried out, we invited eight nurses to participate in the focus group discussion. Unfortunately, one did not find the time to participate, another accepted but did not show up, and hence the focus group discussion consisted of six nurses. The focus group's purpose was to seek a range of views on the competencies gained and the challenges experienced. Table 1 summarizes the characteristics of the participants in the in‐depth interviews and the focus group discussion.

**TABLE 1:** Characteristics of participants included in the interviews and focus group discussion

	In‐depth interview	Focus group discussion
Number of participants	14	6
Number refused to participate	0	0
Number accepted but failed to make it	2	2
Females	11	4
Males	3	2
Interview time	Meantime: 70 min (range 34–126 min)	86 min
Mean years of education related to nursing after high school	3.8 years (range 3–6 years)	3.5 years (range 3–4 years)
Highest level of education (both groups)	Diploma Bachelors Ongoing master studies	
Number of participants with extended responsibilities as in‐charges or matrons	5	3
Mean age of the participant	34.7 years (range 28–42 years)	34.8 years (range 28–39 years)
Mean years of working as a nurse	9.6 years (6–23 years)	‐

### 3.4 Data collection procedures

The semi‐structured, open‐ended interview guides were developed based on the research questions, reading relevant literature, the researcher's experience within the field and formal and informal conversations with key persons in BONEP (see Appendices S1 and S2, interview guides for in‐depth interview and focus group discussion). The interview guides were piloted with one of the Malawian nurses for two main reasons. First, to ensure that the interview questions were culturally and linguistically appropriate and easy to comprehend. Second, to prepare the first author (a novice researcher) for the interview situation. After the pilot interview, some modifications to the guides were conducted. We initially applied the theoretical concept of ‘knowledge sharing’ in the interview guides. However, as we moved forward with the data collection, it became evident that the idea of knowledge sharing had cognitive implications that seemed too narrow to appropriately describe what the nurses were talking about. Competency, as defined in the introduction, seemed like a broader and more suitable concept.

All the interviews and the focus group discussion were conducted face to face, using an audio recorder, in the hospital premises of QECH, except for two participants who were interviewed through Skype. The majority (nine) of the 12 interviews and the focus group discussion were carried out in the hospital's surgical skills laboratory, a quiet and private place with minimal disturbances. The remaining three interviews were carried out in the outdoor surroundings of QECH.

The interviews and the focus group were conducted using the English language as all the nurses are fluent in English. The in‐depth interviews provided most of the data used in the analysis. Towards the end of the field‐work period, the same key themes and perspectives were re‐emerging in the in‐depth interviews. After the point where the data material appeared complete, a few more interviews were conducted, affirming the impression that thematic saturation had been reached (Saunders et al., 2018). The focus group discussion did not add any new themes but instead confirmed what had already been expressed in the interviews.

### 3.5 Data analysis

The study was conducted as the first author's master degree with guidance from two Malawian and two Norwegian supervisors. Verbatim transcription of all the audio recordings was done by the first author, with supervisors' supervision and confirmation. The transcription software F5 was used. The de‐identified transcribed F5 text‐edit formats were converted into Word document format. Manual analysis was done by highlighting essential words and phrases and making comments in the text during the coding and categorizing process. After verbatim transcriptions, the first author developed and iteratively refined the codebook beginning with a priori codes from the interview guide. Initially, 63 codes were identified through open coding and abduction. Open coding can be described as underlining and writing down brackets of the text that appear important (Dahlgren et al., 2004; Maxwell, 2012). Having identified 63 codes, we went back to the data material. A cyclical process of focussing narrowly on the text and re‐reading the material from a more distant perspective was conducted. Through this cyclical process, described as retrogression (Kvale et al., 2015), the 63 codes were reduced to 31. The 31 codes were summarized, and these summaries formed the basis of our content analysis, consisting of four themes, of which three have sub‐themes. Most of the codes were derived from the in‐depth interviews. Analysis of the focus group discussion did not give any new codes.

The question of validity was addressed through all the phases of the research. Within qualitative research, it is well‐known that interviewees may choose to withhold detailed descriptions or embellish them, particularly if the ‘truth’ is inconsistent with their preferred self‐image or if they wish to impress the interviewer (Fielding, 1994). Such considerations raise the issue of whether interviewee‐interviewer characteristics (e.g. demographics) should, at times, be matched (Fielding, 1994). Also, although interviewers may wish to adopt a relatively neutral role, they may inadvertently demonstrate a preference for a particular perspective and, in the process, bias the findings. Therefore, specific attention was given to the threats of reflexivity and bias. During the interviews, respondent validation or ‘member checks’ (Maxwell, 2012, p. 126) was continuously performed to cross‐check with the participant what was being communicated. After the analysis had been conducted, one of the study's key participants read the anonymized results as a form of validation on all the nurses' behalf.

### 3.6 Ethics

The Norwegian Data Protection Services were notified about the research before data collection (project number 60471). The study was exempted from review by the Regional Committee for Medical and Health Research Ethics in Norway. The equivalent committee in Malawi, the College of Medicine Research and Ethics Committee, also exempted the research from review (protocol number 06/18/2416).

All participants were provided with verbal and written information about the study from the first author. It was emphasized that they could withdraw from the study, at any time, without having to give any reason why. Everyone was allowed to ask questions before the in‐depth interviews and the focus group discussion. Moreover, all participants were told that they could reach out to the researcher, or the other members of the research team, at any time after data collection had come to an end. Written informed consent was obtained from all participants before data collection. The data were stored safely according to guidelines at the University of Oslo. How the data were retrieved and stored was mirrored in the participant information sheet.

## 4 RESULTS

**FIGURE 1:**
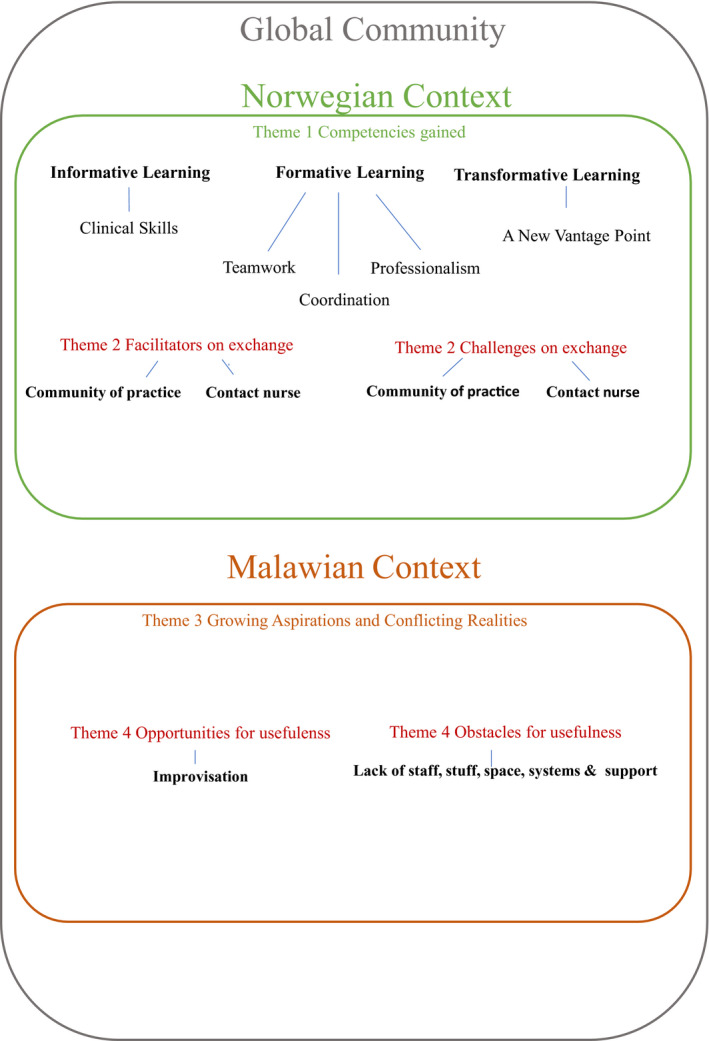
Illustrative Presentation of the Findings

In this section, we present the four main themes, of which three have sub‐themes. First, we explain what competencies the Malawian nurses report to have gained in Norway. Second, we present facilitators and challenges experienced while on exchange at OUH. Third, we outline the perceived usefulness of these competencies upon return to Malawi, and finally, we present opportunities and obstacles upon return to the local context. The four themes will be presented with quotes to support the findings. The quotes are not linked to the participants in any way. To ensure that anonymity is safeguarded, we have given the quotes pseudonyms. We will briefly debate the findings in the result section before the discussion. For an illustrative presentation of the findings, see Figure 1.

### 4.1 Theme 1: Competencies gained

#### 4.1.1 Informative learning‐clinical skills

The Malawian nurses perceived to have gained a variety of clinical skills and competencies, such as improved clinical assessments of patients pre‐, per‐ and postoperatively, to set up a theatre, sterilization of equipment, positioning of patients pre‐ and postoperatively, the use of checklists and guidelines, management of external ventricular drains, observations related to increased intracranial pressure, knowledge on fluid balance and nutrition, bedside nursing, knowledge of the Glasgow Coma Scale and on a variety of neurosurgical diagnosis. One of the nurses expressed it this way;

I also learned that when it comes to nursing, it ´s not only about medication. It ´s about the actions and the interventions that you're doing without medications. You have to look at the fluid balance, and you have to look at the nutrition. You have to look at making the patient psychologically stable. All those things are more powerful than just giving the patient medication. And the other thing that I learnt was the use of multidisciplinary teams (Judy, IDI).

Another nurse said:

[…] some of the conditions, the neuro conditions I had not met, because the time I went to Oslo, I didn't have much knowledge, like in neuro, neuro conditions, how to manage them. Of course, we learnt some topics in college about how to manage a patient with brain injury, with just in passing. But I didn't have the experience to care for those kinds of patients and manage them. So, I can say I learnt a lot in terms of knowledge on the different conditions that I saw in Oslo, and how to manage, the nursing management, and even the surgical management, how the patients are managed (Christine, IDI).

#### 4.1.2 Formative learning—teamwork, coordination and professionalism

All the participants expressed that interdisciplinary teamwork, including communication and collaboration, were important values and competencies that they had observed and learned about while on exchange. Most of the participants also mentioned the way hospital work was organized and administered in Oslo. All the participants believed that neurosurgical nursing had become part of their professional identity and that the exchange had allowed a movement from generalists to specialists. Many conveyed that the exchange had created a sense of strengthened professionalism resulting in an improved work ethic.

The other thing I learnt was teamwork, there was a lot of teamwork. Teamwork in terms of doctors, nurses, everyone coming together, and I think, respect each other. That ´is also one of the things (Judy, IDI).

Keeping time and being punctual to carry out their professional responsibilities were perceived as necessary, facilitated by a well‐functioning public transit system. One participant described the transport system in Malawi and then compared it to Norway.

Oh, you have to walk to the station and then wait for the minibus, and it takes time, maybe the minibuses are coming, but they are full, and there ´s also trafficking, the traffic jamming, the roads, and you find yourself coming to work and reaching work at the workplace very late. Whereas there (referring to Norway) you have, most of the time you are on time, and you come before the time at work, because the transport system is very good there (Carin, IDI).

#### 4.1.3 Transformative learning: a new vantage point

The exchange was the first time to leave Malawi for 11 of 14 nurses included in the study. However, all shared common views concerning the transformative nature of the exchange. The exchange and the competencies gained on exchange contributed to what all the nurses considered a transformative and mind‐opening learning experience, both personally and professionally. In general, western European digital life experience and a highly specialized hospital setting in specific seemed to merge into a life‐changing event. The participants expressed that living and working in a new environment, very different from the habitual setting, led to a shift or a transition of perspective and a change of attitude that all the nurses believed to be profound and persistent.

[...] the exchange is not all about the skills, the hands‐on skills that you are going to learn. To me, I found the skills are also about changing your mindset, which I found to be a very important component (Peter, IDI).

Another nurse described it this way:

[...] before I went to Norway, I had some perceptions, all those building blocks in my mind about whatever we do, that whatever we do here is ideal. But when I went to Norway, I was changed, because, my attitude changed (Paul, IDI).

### 4.2 Theme 2: Facilitators and challenges on exchange

#### 4.2.1 Belonging to the same community of practice

A majority of the nurses perceived the beginning of the exchange in Norway to be challenging. However, this changed after a while. For some, it changed after a few weeks, for others only at the end of the stay. Most participants believed the shift from passive observers in the periphery towards active participants happened around 3 months. Belonging to the same community of practice, sharing the same profession was believed to facilitate this dynamic process of being more actively included in clinical hands‐on patient work. The expression ‘we are all nurses’ was repeatedly mentioned.

At first, it was just observing, but then when I was integrated into the everyday routine, then I would be able to practice on the patient, with the supervision of course (Susan, IDI).

During the clinical training, the availability and presence of a contact nurse reinforced the perception that nurses belong to the same community of practice globally. During the 6‐month exchange period in Norway, the Malawian nurses were assigned at least one contact nurse during the clinical training and who supervised their daily duties. Having a contact nurse was regarded as beneficial to the learning process as this enabled a dynamic personal and professional relationship to develop based on trust.

The project should at least make sure that there should be two or three contact nurses for an individual because that helps that person to know, to help you know the things that you don't know, to orient you to some other things, and also, the learning process can progress (Carin, FGD).

Another nurse communicated it this way:

Yeah, because it was even the first, the first week. Yeah, they started involving me in everything (Paul, IDI)

However, not all nurses who participated in the exchange programme benefited from a contact nurse's role. Personal challenges related to the absence of a contact nurse were feelings of loneliness, sadness and not being included in the work environment.

[...] and especially on those days, the specific days when the contact nurse was not around, and if you meet the person who is not so willing to, like work with you. It would feel so lonely (Mary, IDI).

Professional challenges mentioned were the inability to make progress, start all over again with a new contact person and being left out of the clinical activities. There were disparities related to the degree of closeness established with the contact nurse, which seemed to be associated with the degree of continued contact. A few participants had established sustainable and solid relationships that were existing up to date.

#### 4.2.2 Remaining an observer on the periphery

Shadowing a contact nurse also unearthed negative feelings as some Malawian nurses felt that they were standing outside of their profession looking in. In addition, being a passive observer on the periphery triggered the sense of not being able to participate fully. A few participants compared the role of a passive observer to the feeling of being a student again, which for some was regarded as constraining, as exemplified by the following quotes.

You know when you ´re a professional already in work, and it ´s different when you ´re a student. When you are a professional, you would like to see yourself preoccupied with the work. Otherwise, it ´s boring, if you just go, and have to be a watcher from 7.30 to 3.30, it is a little bit boring (Peter, IDI).

[…] there's no contact nurse for you for that day, and you go into the ward, you don't know who you are going to go with, right? So, some nurse is willing to go with you but does not understand what you can do and what you are capable of doing. So, she just sees you around with a certain contact nurse who has not been there for that day. So, she'll just take you, and be with you and let you observe (Mary, IDI).

### 4.3 Theme 3: Usefulness of the competencies gained on exchange

#### 4.3.1 Growing aspirations and conflicting realities

All the nurses regarded the competencies gained on exchange to be helpful and valuable upon return to their local clinical context in Malawi. Furthermore, all participants perceived BONEP and the exchange programme to be beneficial, not only for themselves as individual nurses but also to the patients and QECH as an institution. Some nurses told patient stories, illustrating how the quality of neurosurgical care had improved over the years and connected it to the competencies gained on exchange. For example, one nurse, who had been on the job training in Oslo during the first years of the programme, told a story depicting her experience with a patient's death in QECH before her exchange visit. She regarded the lack of knowledge related to postoperative neurosurgical assessments as a contributing reason for why the patient had died. The following quote describes what happened during the first night after the patient had undergone a successful craniotomy.

The nurses didn't know how to handle that patient because honestly, everyone was blank, and it's just unfortunate that the patient died (Judy, IDI)

Also, for most nurses, the experience of life outside Malawi in a high‐income European country with raised hospital standards and living conditions marred the enthusiasm to return home. Returning to Malawi was filled with mixed emotions, including new aspirations and hopes for the future. However, without specific opportunities to fulfil these new aspirations and hopes for the future, some nurses were left feeling demoralized, sad and demotivated.

[…] coming back from Norway where transportation is there, where the internet was so good, and almost everything, the living standard is high, and coming back to Malawi it was like a shift (laughter), going back again, having electricity interruptions, water interruptions. So the first weeks it was not so comfortable (Christine, IDI).

Another nurse stated it this way:

It was not really like, you know when you are in‐between, you're not excited, you're not really like too bad, you're just in a, in a state where you're just muted, you know. You don't know what to do. So that was the main feeling that I had when I came back (Juliette, IDI).

### 4.4 Theme 4: Opportunities and obstacles for usefulness in local context

#### 4.4.1 Improvisation as a premise for utility

All participants regarded the competencies gained to be valuable upon return to QECH. However, everyone acknowledged that improvisation was necessary for the skills and competencies to be usable in Malawi. Improvisation can be regarded as the resilient task of tailoring skills, needs and resources to fit the local context. The word most often used to describe this process of transforming or adapting to local context was improvisation. Sometimes, creativity or innovation was used. In the following quote, the nurse explains how to measure the level of resistance of an external ventricular drain in QECH. External ventricular drains are applied in neurosurgery when there is a need to drain cerebrospinal fluid. It is typically used to avoid hydrocephalus and increased intracranial pressure (Korinek et al., 2005).

[…] for us, we have to measure it using a local way. That means we have to measure it using a ruler […] we use it to balance up that it should be 15 centimetres (Julia, IDI)

Two other nurses communicated it like this:

We have to take from what is new from Norway and then fix into our setting and see if it can work (Ann, IDI)

[…] we need to have the suction tubes, […] instead of just taking the sterile ones, the disposable ones, we don ´t have enough of them, so we just reuse them. We dip them in cidex (a disinfecting solution) for some minutes, and then we use them. So, it ´s like, instead of just having those already sterilized disposable ones, we reuse them (John, IDI).

#### 4.4.2 Lack of resources and support

The main obstacle experienced upon return to Malawi concerning the utility of the competencies gained was the lack of resources. While it could be anticipated that the scarcity of material, human and coordinative resources would represent an obstacle, many participants also mentioned the system's lack of support upon return. Twelve of the 14 participants interviewed expressed it as a gap between the support services provided for on exchange versus on return. Some described it as ‘coming back to nothing’.

[…] you need a system to support the participants, to implement whatever they have learnt from the exchange visit. Because, without support, the support of the system, I think it has shown difficult to implement some of the things that I think we learnt from the exchange (Peter, FGD).

The following participant describes the lack of resources and poor salaries:

I have a lot of patients to work with…I don't have resources, I receive less money, why should I bother myself to…so it takes someone passionate to stay about the profession (Mary, IDI).

## 5 DISCUSSION

To our knowledge, this is the first study to investigate what competencies Malawian nurses report to have gained during the 6‐month job exchange in Norway and how these competencies were perceived to be valuable and applicable in a Malawian context. We specifically examined facilitators and challenges faced on exchange in Norway and upon return to Malawi.

### 5.1 Competencies gained in Norway

Following Epstein and Hundert, and in line with the World Health Organization (World Health Organization, 2016), professional education should be valued by its transformative ability (Epstein & Hundert, 2002; Frenk et al., 2010). However, an attractive feature to note is that Epstein and Hundert define competence as ‘habitual’ (Epstein & Hundert, 2002, p. 226). The results of this study suggest that indeed the very absence of what is seemingly habitual, that is, the move from Malawi to a different context in Norway, permitted an experience regarded as transformative to occur. Competence, therefore, seems to be composed of both the habitual, consistent and repeated encounters within a field, often achieved through years of experience contributing to making knowledgeable clinical experts (Epstein & Hundert, 2002; Frenk et al., 2010), as well as the opportunity to contest and confront these very competencies and perceptions. This confrontation allows new perspectives to evolve and previously agreed upon assumptions to be renegotiated (Frenk et al., 2010). Related to this is the perception that the exchange led to a shift from generalists to specialists. There are no critical care educational programmes in Malawi for nurses (Gundo et al., 2019). The exchange, therefore, provided the participants with a unique opportunity to get specialist training.

Belonging to and actively participating within a shared community of practice was identified as the most significant facilitator during the exchange. Lave and Wenger's theory on situated learning also acknowledges the nature of transformative learning and suggests that knowledge, understanding and the context in which learning occurs are closely connected. Moreover, common communities of practice allow for learning and competence improvement (Lave & Wenger, 1991). In line with Lave and Wenger, and Epstein and Hundert, this study justifies the notion that it is fruitful to see competence building as part of social interaction and that learning and competence improvements include the formation and growth of meaningful social relationships (Lave & Wenger, 1991). Furthermore, the gradual process of moving from the periphery into the centre includes the notion of change, from observing towards acting. Therefore, the empowering quality of learning and competence building involves changing from a ‘passive’ observer to a hands‐on ‘active’ participant.

### 5.2 The usefulness of competencies gained in Norway upon return to Malawi

While the participants in this study insist that the competencies gained in Norway are helpful in a Malawian context, they also express that the exchange resulted in growing aspirations connected to living a life in a context perceived to be embedded with more opportunities and hopes for the future. The return to the home community represented a reunion with a context in which the competencies and the opportunities and hopes gained were perceived as challenging to implement and fulfil. While the participants had changed and transformed during the exchange, not only regarding the improvement of clinical and professional competencies gained but rather as a ‘person‐in the world’ (Lave & Wenger, 1991, p. 52), the setting that they returned to, in many ways had not. This finding confirms a main argument in the literature presented, namely the tight relationship between the competence of a person, the tasks needed to be performed and the surrounding clinical context (Epstein & Hundert, 2002; Frenk et al., 2010; Lave & Wenger, 1991; Meara et al., 2015).

Theories connected to transformative learning and transformation often involve notions about shifts or change (Frenk et al., 2010; Lave & Wenger, 1991; Mezirow, 2003). However, the growth and evolution that the Malawian nurses experienced during the exchange were not permanent but instead temporary. In other words, the experienced transformation, evolving from the 6‐month exchange, contributed to opening up doors to a novel and high‐income western world, growing aspirations about how life could be lived, and increased competencies related to neurosurgical nursing. Yet, upon return to the local clinical context and the conflicting realities between Norway and Malawi, many participants described a feeling of demotivation and even sadness. Having been away for 6 months, the return proved a sober reminder of a life generally perceived as more challenging and with fewer opportunities than the life experienced in Norway.

The above finding underlines another key element about competence building, namely that it extends beyond the individual capacities of single health workers. It points to the need to address quality improvements at a system level (Kruk et al., 2018). While the health workforce constitutes a vital part of a well‐functioning health system, it is not the only part. Infrastructure, the supply of medicines and technologies, funding and evidence‐based policies are other components. Nevertheless, the health workforce in low resource settings faces several obstacles related to access to necessary resources (Wendland, 2010, 2012). Besides, this study's participants described a desire for more support, a finding that corresponds with research done among nurses from Lilongwe in Malawi (Maluwa et al., 2012). In line with the literature, the participants argued that improvisation and creative use of resources are necessary to overcome these obstacles (Meara et al., 2015; Scott et al., 2018; Wendland, 2010). It is, therefore, suitable to return to the definition by Epstein and Hundert of competence. Not only is competence defined as habitual, but it is also characterized as judicious. However, what seems to be judicious varies across clinical contexts of care. Judicious neurosurgical nursing competence is not a constant concept but rather a highly flexible one that needs to consider the contextual barriers and facilitators at the point of care.

### 5.3 Strengths and limitations

The data collection was carried out in the setting where the Malawian nurses work, in QECH in Blantyre, Malawi. The closeness to the context and the field contributed to valuable nuances in the analysing process. The first author is a nurse who has been involved in Malawian nurses' clinical supervision in BONEP. On the one hand, her association with the programme represents both a potential strength and limitation. It might have improved the communication and perceived feelings of trust and familiarity within the study population and increased the degree to which the participants could express criticism towards the programme. On the other hand, it could make the Malawian nurses more prone to express opinions unilaterally positive by nature. Feelings of gratitude or loyalty could threaten the degree to which the participants experienced the ability to speak freely and honestly, a phenomenon known as reflexivity. However, the impression is that the Malawian nurses spoke about both positive and less positive aspects of BONEP, as demonstrated in the results section.

Finally, another limitation is language. Although all participants are fluent in English, we cannot exclude that views might have been better expressed if the interviews and the focus group were conducted in the Chichewa language.

## 6 CONCLUSION AND SUGGESTIONS FOR FUTURE RESEARCH

It could be viewed as a paradox that while training and competence building in the literature often is characterized as inextricably linked to the environment in which it is to be performed, and therefore encourages competence building in own local environment (Frenk et al., 2010; Lave & Wenger, 1991; Meara et al., 2015; Napier et al., 2014), this study has shown that indeed an alteration of the habitual setting and environment can lead to a change of perspective that is viewed as beneficial and sustainable. It, therefore, seems fair to acknowledge the dichotomous nature of competence building within the framework of an institutional health partnership. On the one hand, competencies need to be tailored to the context where they will be used and practised. On the other hand, transformative learning seems to have been achieved through the institutional and clinical exchange away from the habitual context. This suggests that competence building not necessarily and primarily should be done in the local home community. While local training might be more effective in finding practical solutions and coping with problems and challenges at the point of care, this study shows that an exchange programme can result in changes of mindsets and a renegotiated view of the quality of care. In other words, crosscutting clinical contexts of care has the potential of contributing to competence building that involves transformative learning and includes the whole ‘person‐in the world’ (Lave & Wenger, 1991, p. 52). However, the study also suggests that such a transformation in attitudes and mindsets have limited effects if structural and resource constraints are not addressed. Examining whether transformative learning also led to transformative clinical practice and improved patient outcomes will need to be further investigated in the future.

## CONFLICT OF INTEREST

No competing interests are disclosed.

## AUTHOR CONTRIBUTIONS

CGA: Conceptualization, data collection, analysis and writing of the manuscript. PDK and RJP: Conceptualization, assistance in the analysis, editing and revising the manuscript. JS and CM: Conceptualization and editing manuscript. LMT: Writing and revising the manuscript. All the authors have agreed on the final version of the manuscript.

## ETHICAL APPROVAL

The Norwegian Data Protection Services were notified about the research before data collection (project number 60471). The study was exempted from review by the Regional Committee for Medical and Health Research Ethics in Norway. The equivalent committee in Malawi, the College of Medicine Research and Ethics Committee, also exempted the research from review (protocol number 06/18/2416). Written informed consent was obtained from all participants before data collection.

## Supporting information

Appendix S1

Appendix S2

## Data Availability

This study was conducted as part of the first author's master thesis in International Community Health at the University of Oslo. All informants gave consent for participation in the study and were informed in the written information sheet about the ambition of publishing an article in a peer‐reviewed journal. The data sets used and analysed are available from the corresponding author upon reasonable request.
