# Supplementary material for: Improving competencies and skills across clinical contexts of care: a qualitative study on Malawian nurses' experiences in an institutional health and training programme
Source: Nurs Open. 2021 Aug 6;8(6):3170–80. doi: 10.1002/nop2.1030 (PMC8510767; doi:10.1002/nop2.1030)
Supplement: Supplementary file 1 — Appendix S1 [file NOP2-8-3170-s001.docx]

# **Appendix 1- Interview guide in-depth interview**

## Questions concerning participants’ background

1. I am interested in knowing a little bit about you and your background. Please tell me about why you decided to become a nurse?
2. Could you tell me about what kind of education and work experience you have?
3. Please walk me through a day at work in Queens, for example how was yesterday (or the last day you worked)?

-Would you consider this a typical day? In what way or in what way not?

-Which ward do you work in? What tasks do you have?

-What time do you start and when do you finish?

-How many patients do you normally have?

1. If you have a day at work were you feel that there is something you do not really know how to do, would you consider your work environment to be of the kind that you could ask colleagues for help and that you would share experiences and skills?

-Do you consider nursing to be teamwork, and in what way/why not?

-How do you understand knowledge sharing?

## Questions concerning the participants exchange period

1. When did you get involved in the Blantyre-Oslo Neurosurgical Exchange Program, and when were you on exchange to Norway?
2. Tell me about how you experienced the exchange period?

-How did you experience the beginning of the stay, coming to work in a Norwegian hospital?

-Did this last for the whole stay or did it change in some way. How did it change?

1. Please tell me if there were many barriers that you encountered related to the work in the hospital, and if and how you managed you overcome them (in the beginning, the middle and the end of the stay if it makes it easier to divide the time).

-Did you experience any barriers of more personal character that you can share with me?

-How did you feel staying away from your family for six months?

1. In your opinion, what is the difference being a nurse at Queens in contract to at Oslo University Hospital?
2. What did you learn in Oslo that you did not already know from before?

-Just how did this happen? Hands on by the patient, by the working station, by reading, in interaction with colleagues, please give me one or two examples.

1. We talked a bit about how to share experiences and skills earlier (question number 4). Did you ever experience that people in Norway were interested in how you do things in Malawi?

## Questions concerning experiences after the exchange period

1. How did you experience the return to Malawi?
2. You mentioned that you learned xxx (touch upon what the participant has said previously) in Norway. Was this useful upon return to Malawi, could you apply it? Have you continued to feel this way?
3. Are there any challenges in regard to making (or translating) what you have learnt in Norway to be useful in a Malawian context?

-Other than what you have already mentioned, are there any other barriers that I should be aware of, that you would like to mention?

1. In spite of the lack of resources (if they mention this), do you still think you contribute to sharing what you have learnt from your exchange in Oslo to your colleagues in Malawi?

-In what way?

1. As I have understood it, there is a two-year lock-time after the exchange period, upon return to Malawi. Would you like to stay within this field, also after the lock-time?

-Has neurosurgical nursing become part of your professional identity?

1. How do you picture the neurosurgical services at Queens in 5 years (end of the project)?

-What do you think the program has given you?

-How do you think the program has benefitted Queens?
