# Supplementary material for: Improving competencies and skills across clinical contexts of care: a qualitative study on Malawian nurses' experiences in an institutional health and training programme
Source: Nurs Open. 2021 Aug 6;8(6):3170–80. doi: 10.1002/nop2.1030 (PMC8510767; doi:10.1002/nop2.1030)
Supplement: Supplementary file 2 — Appendix S2 [file NOP2-8-3170-s002.docx]

# **Appendix 2- Interview guide for focus group discussion**

1. How did you get involved in the neurosurgical program?
2. Was the job-exchange in Norway as you expected?

-In what way/in what way not?

-How did you experience the FK week before and after the exchange?

1. When you were in Oslo, did you have one or two specific nurses that you followed during the exchange period, or did this change from day to day?

-If it did vary, how was this experienced?

-If it did not vary, how was this experienced?

-Did you feel that there was a progressive plan during the exchange or was it more like on a day-to-day basis?

1. I am interested in understanding more about how you felt you could participate in the everyday work environment throughout the exchange period?

-Was there a gradual increase in how much you participated in the hands-on work and the interaction with both patients and colleagues or did this remain the same from the beginning to the end?

-Can you give me examples or tell me about episodes that describe this?

1. Did you feel integrated in the work environment?

-Did you feel you could ask for help or ask questions when things were unclear?

-Did the Norwegian colleagues ask you any questions about your everyday work in Malawi or how you used to manage things?

1. You are all experienced nurses from Malawi. Did the work experience add anything to you professionally?

-Did you feel you learnt things you did not know from before?

-Please give me concrete examples, in what contexts was this experienced?

-Did it add anything personally? In what way?

1. Upon return to Malawi, how did you experience that the skills or competence that you gained in Oslo could be useful in your everyday work environment?

-How did you manage to use the knowledge/skills/competencies from Oslo in your own local context?

-Could you use what you had learnt just like that, or did you have to “translate” it in some way to make it applicable to the Malawian context?

1. Did you feel that the skills and the competencies were integrated enough for you to be able to share it with others upon return? For example, management of EVD, monitoring ICP, handling central lines, pain management or other areas where you might have gotten some experience?
2. What facilitated this process of sharing your experience from Oslo with colleagues in Malawi?
3. What did you experience as barriers to this process?
4. How do you understand the term knowledge sharing?

-Do you consider nursing to be team-work? Do you ask each other for help and support each other in the important work that you do?

1. In what way do you think the program has benefitted your own hospital, Queens? How do you think neurosurgery will develop in the future years to come? What are the main challenges? How can your experience contribute?
